# Language and communication development in preschool children with visual impairment: A systematic review

**DOI:** 10.4102/sajcd.v62i1.119

**Published:** 2015-10-16

**Authors:** Renata Mosca, Alta Kritzinger, Jeannie van der Linde

**Affiliations:** 1 Department of Speech Language Pathology and Audiology, University of Pretoria, South Africa

## Abstract

**Background:**

Language and communication difficulties of young children with visual impairment (VI) are ascribed to intellectual disability, multiple disabilities and autism spectrum disorder (ASD) rather than their sensory impairment. Consequently, the communication difficulties of children with VI may have been underestimated and undertreated.

**Objectives:**

This report aims to critically appraise recent peer reviewed literature relating to communication and language development in children with VI.

**Method:**

A systematic search of the literature (2003–2013) was completed using the PRISMA guidelines, and primary and secondary search phrases. Nine publications were reviewed in terms of the strength of recent evidence. Thematic analysis was used to describe the early language and communication characteristics of children with VI.

**Results:**

All the selected articles (*n* = 9) were from developed countries and participants from seven of the studies had congenital VI. Five of the studies received an evidence level rating of III while four articles were rated as IIb. Two main themes emerged from the studies: early intervention, and multiple disabilities and ASD. Language and communication development is affected by VI, especially in the early stages of development. Speech-language therapists should therefore be included in early intervention for children with VI.

**Conclusion:**

Recent evidence on the early language and communication difficulties of children with VI exists, but children in developing countries with acquired VI appear to not be investigated. The identified language and communication developmental characteristics may assist speech-language therapists to build a knowledge base for participation in early intervention for young children with VI and their families.

## Introduction

The impact of visual impairment (VI) on the communication development in young children has been underestimated and undertreated (House & Davidson, 2000; James & Stojanovik, 2007). Underestimation and undertreatment may be because communication difficulties in children with VI are ascribed to intellectual disability and autism spectrum disorder (ASD; House & Davidson, 2000) or multiple disabilities (Chen, 2001) rather than VI. Another reason may be that speech-language therapists are not trained to treat this population as their main focus of training with regard to sensory impairment is on communication delay associated with hearing impairment (James & Stojanovik, 2007).

Early research on children with VI focused more on general development (Carvill, 2001; Davidson & Harrison, 2000; Good, Jan, Burden, Skoczenski & Candy, 2001; Prechtl, Cioni, Einspieler, Bos & Ferrari, 2001) than on communication difficulties. Communication-related studies were predominantly descriptive and mostly relied on expert opinion (Chen, 1999; Goldware & Silver, 1998; Tedder, Warden & Sikka, 1993), whereas the current trend in research is towards a high level of evidence. There is a need to review recent research to examine the strength of the evidence and to describe language and communication development in young children with VI.

Since the visual system is complex and the causes and effects of VI are numerous and intricate (Holte *et al.*, 2006), children with VI form part of a heterogeneous population. Approximately 70% of children with VI present with multiple disabilities (Chen, 2001), and there are more than 80 known genetic and chromosomal syndromes that may result in deafblindness (Holte *et al.*, 2006). The Joint Commission on Infant Hearing therefore endorses the ophthalmological assessment of all infants with confirmed hearing loss (Blumsack, 2009). Multiple disabilities affect clinical decision-making during assessment and diagnosis as the characteristics of disorders may mask or mimic each other (House & Davidson, 2000). For example, the social communication difficulties of children with VI may be mislabelled as autistic tendencies (*ibid*). Information is needed to help identify and improve the understanding of language and communication characteristics in young children with isolated VI and those with VI as part of multiple disabilities.

VI may affect the play, motor, cognitive, social and communication skills of young children (Chen, 2001) as typical development occurs through unrestricted interaction with the environment (Glass, 2002; Owens, 2005). Despite complexity and diversity within the population, children with VI are unified by a significant absence of visual experiences that shape development. Developmental difficulties of young children with VI and the nature of the development of the visual system suggest the need for intervention within the first 12 months of life (Davidson & Harrison, 2000). Increased information about language and communication development in young children with VI may improve early identification of communication difficulties, assist in goal setting and draw attention to the need for early communication intervention for this population.

Children with VI, and especially those with additional impairments, may require direct language instruction in order to develop language skills (Chen, 2001), highlighting the need to include speech-language therapists in the early intervention team for children with VI. Early intervention, as an evidence-based strategy (ASHA, 2008; SASLHA, 2011), is known to augment young children’s development and promote better long-term functional outcomes for both the child and the family (Fazzi, Signorini, Bova, Ondei & Bianchi, 2005). There is a need to review recent research to examine the strength of the evidence and to describe language and communication developmental characteristics in young children with VI. This will assist speech-language therapists to build a knowledge base for participation in early intervention for young children with VI and their families.

The research questions posed in this systematic review were twofold: What is the strength of recent research evidence regarding early language and communication development skills of children with VI, and what are the children’s characteristics in these developmental areas?

## Method

### Study design

A systematic review was conducted to answer the research questions posed. The PRISMA (Preferred Reporting Items for Systematic Reviews and Meta-Analyses) statement (Moher, Liberati, Tetzlaff & Altman, 2009) was used to structure the systematic review. The PRISMA checklist helps ensure the transparent and complete reporting of systematic reviews (*ibid*). This research project received ethical clearance from the Research Committee of the Department of Speech-Language Pathology and Audiology, and the Research Ethics Committee of the Faculty of Humanities of the University of Pretoria.

### Study inclusion criteria

The inclusion criteria comprised of articles pertaining to communication, language and speech development and characteristics thereof in young children (birth to five years) with any form of VI. VI is defined as the loss of any aspect of vision that diminishes the ability to see. The International Classification of Diseases (ICD-10, Update and Revision 2006) identifies the following ranges of vision: normal (equal to or better than 20/70), moderate (20/70–20/200) and severe VI (20/200–20/400). Moderate and severe VI are grouped as low vision. Blindness is categorised over three ranges: blind (20/200–20/1200), blind with light perception and blind with no light perception (WHO, 2012).

No limit was placed on the type of study selected. Based on relevance to the subject field, the following electronic databases were searched: MEDLINE, Scopus, PsycINFO and PubMed. Since the concepts communication, language and speech are used interchangeably in databases, these three concepts were coupled with development or characteristics in separate searches of each database. The main search phrases were ‘communication development’ and ‘communication characteristics’, for example, ‘communication development in children with VI’ and ‘communication characteristics in children with VI’. These phrases were used in two respective searches in each of the four databases. For the related search phrases, ‘language’ and ‘speech’ replaced ‘communication’ as the main phrases. A total of 24 searches were conducted across four databases. This electronic search strategy, limited to 2003–2013, resulted in the retrieval of a total of 1661 articles from the initial search. An age limitation of birth to five years was then applied. The last search was run in November 2013.

### Study selection

**FIGURE 1:**
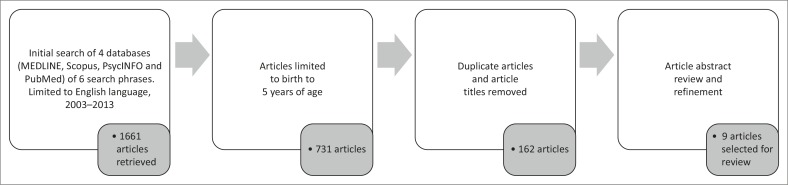
Review phases used to identify articles for inclusion.

All the English language article titles were reviewed and duplicate articles were removed (162 articles remained). The abstracts of the selected articles were then reviewed. The remaining nine articles meeting the inclusion criteria and discussing communication development and/or characteristics thereof were selected (see Figure 1). The full articles were reviewed to identify the communication, speech and/or language development or characteristics of children (birth to five years) with VI. To avoid bias, consensus was reached between the three authors regarding the final inclusion of articles.

### Data collection process and data items

Data collection took place by studying each article and extracting information to form summaries of the articles, displayed as mind maps. In terms of data items, information was collected from each article on:

1. Characteristics such as title, authors, year of publication, country where study was conducted, participant age range and number, method, level of evidence and visual status of participants.
2. The developmental communication characteristics of participants detailed in the article, including communication, language and speech development.

As it is widely accepted in the field of speech-language therapy, the ASHA level of evidence rating scale (ASHA, 2004) was used to categorise the articles in the final selection according to the level of evidence. These ratings are discussed within the results section of this article.

### Risk of bias in selected studies

The criteria for the assessment of risk of bias were modified from the Cochrane Collaboration’s tool for assessing risk of bias (Higgins, Altman & Sterne, 2011). The following criteria were included: steps taken to avoid selection bias, blinding of participants, personnel or outcome assessors (when information about the study that might lead to bias in the results is concealed), the presence of control groups or tools, the involvement of more than one clinician in evaluations, inter-rater agreement and the use of validity, internal item consistency and/or reliability testing. None of the selected articles specifically described an assessment of risk of bias other than providing statements pertaining to possible bias. Decisions on the risk of bias were made by consensus between the authors.

### Data analysis

Thematic analysis (De Vos, Strydom, Fouché & Delport, 2005) was used to organise the information extracted from the selected studies and to synthesise results. Main themes were identified within the data and sub-themes were assigned from the study outcomes.

## Results and discussion

### Study characteristics

The characteristics of the nine selected articles are presented in Table 1.

**TABLE 1:** Summary of articles selected for review.

Title	Authors, year and country where study was conducted	Participant age range	Number of participants, including controls	Research method	Level of evidence (ASHA 2004)	Visual characteristics of participants
Development and characteristics of children with Usher syndrome and CHARGE syndrome	Dammeyer, 2012, Denmark	Usher syndrome: 3–17 years CHARGE syndrome: 0–15 years	Usher syndrome: 26 CHARGE syndrome: 19	Survey using medical case records and deafblind consultants	III	Congenital and progressive deafblindness
The dynamic landscape of exceptional language development	Peltzer-Karpf, 2012, Austria	Four studies: 6–10 years, 5–11 years, 18 months-8 years Main case: 18 months-3 years	72	Longitudinal study over 18 months with control groups	IIb	Congenital VI
Development of a vocabulary of object shapes in a child with a very-early-acquired visual agnosia: A unique case	Funnell & Wilding, 2011, England	Study conducted between 2 and 14 years	1	Longitudinal design over 8 years, single case study with retrospective data included	III	Acquired visual agnosia
Developing a schedule to identify social communication difficulties & autism spectrum disorder in young children with visual impairment	Absoud *et al.*, 2011, England	1 year 9 months-6 years 11 months	23	Comparative design for observational tool development	IIb	Congenital VI
Social communication difficulties and autism spectrum disorder in young children with optic nerve hypoplasia and/or septo-optic dysplasia	Parr *et al.*, 2010, England	10 months-6 years 10 months	83	Longitudinal study over 32 years, retrospective case notes review with between subject comparison	III	Congenital VI
Differentiating characteristics of deafblindness and autism in people with congenital deafblindness and profound intellectual disability	Hoevenaars-van den Boom *et al.*, 2009, The Netherlands	7–28 years, developmental age of less than 24 months	10	Comparative design for observational tool development	IIb	Congenital deafblindness
Communication in the early stage of language development in children with pCHARGE syndrome	Peltokorpi & Huttunen, 2008, Finland	Three participants: 1.4, 3.9 and 8.4 years	3	Descriptive multiple single case study design with survey elements	III	Congenital deafblindness
Early communication in dyads with visual impairment	Rattray & Zeedyk, 2005, England	6–18 months	5 dyads (n10)	Longitudinal, comparative design over 1 year with static four-group design	IIb	Mother or child or both had VI
Emotional status and development in children who are visually impaired	Ashkenazy *et al.*, 2005, Israel	0–5 years	74	Longitudinal, comparative design over 18 years	III	Congenital VI

Selected articles in Table 1 ranged from 2005 to 2012 and were all conducted in developed countries. However, it is estimated that there are 285 million people in the world with VI (WHO, 2014), 90% of which reside in developing countries (WHO, 2010). Seven of the nine articles described participants with congenital VI. Of the remaining two studies, one investigated VI acquired at eight weeks old and the other study did not provide the aetiology of VI in the mother-child dyads. The prominence of congenital VI in the study sample may be characteristic of current research in developed countries. Conversely, Gilbert and Foster (2001) state that VI in developing countries is usually acquired.

The cumulative participant age range for all nine articles was birth to 28 years. Participants older than five years were included in the studies as mental disability resulted in functioning below a developmental level of five years of age. The number of participants varied widely across each selected study, from one to 83 individuals. Five of the selected articles were longitudinal studies (Ashkenazy, Cohen, Ophir-Cohen & Tirosh, 2005; Funnell & Wilding, 2011; Parr, Dale, Shaffer & Salt *et al.*, 2010; Peltzer-Karpf, 2012; Rattray & Zeedyk, 2005) of which three utilised the most participants of the nine studies. It is remarkable that such large samples could be recruited as VI is a low incidence disability and participant attrition is a disadvantage of longitudinal research. In the study with the greatest number (*n* = 83) of participants (Parr *et al.*, 2010), this was achieved through a retrospective review of case notes collected over 32 years. In the study by Peltzer-Karpf (2012), a meta-analysis from four studies was conducted. Participants in the study by Ashkenazy *et al.* (2005) were recruited from a specialised unit for children with VI in a large child development centre.

The strength of recent research evidence of the early language and communication skills of children with VI shows that five of the articles (Ashkenazy *et al.*, 2005; Dammeyer, 2012; Funnell & Wilding, 2011; Parr *et al.*, 2010; Peltokorpi & Huttunen, 2008) achieved a level III rating. These studies were either comparative investigations, retrospective and prospective case studies, or survey designs. The remaining four articles received a higher rating of IIb as they were identified as well-designed quasi-experimental studies. Two studies (Absoud, Parr, Salt & Dale, 2011; Hoevenaars-van den Boom, Antonissen, Knoors & Vervloed, 2009) developed assessment tools. Peltzer-Karpf (2012) made use of neuroimaging to compare study group results, and Rattray and Zeedyk (2005) conducted a longitudinal study with a static four-group comparative design.

None of the articles achieved a level of evidence of IIa and above. It appears that controlled studies with randomisation may advance research in this field. By using randomisation, with a representative sample, comparisons could be made between participants with various conditions presenting with VI or between age-matched peers without VI (De Vos *et al.*, 2005). This would assist in singling out the impact of VI on language and communication development. However, as children with VI are a diverse population, randomisation is a challenging task. There are many possible contributing factors besides VI that can influence developmental functioning. Identified factors include multiple disabilities, extended hospitalisation (and therefore environmental deprivation), age of identification, economic status, caregiver behaviour, intellectual ability and behavioural difficulties. Although most of the study designs and methods still represent the lowest level of evidence, the difficulty of conducting research on children with VI should be considered. There appears to be a move to more sophisticated designs and objective methods such as the neuroimaging used by Peltzer-Karpf (2012), which may provide a new avenue in research. In summary, the validity of recent research on the language and communication characteristics of children with VI appears to be increasing in level of evidence.

Four (*n* = 9) of the studies (Absoud *et al.*, 2011; Dammeyer, 2012; Hoevenaars-van den Boom *et al.*, 2009; Parr *et al.*, 2010) highlighted the lack of assessment tools, and Peltokorpi and Huttunen (2008) required the modification of two tools to exclude visually loaded items. This can result in subjective, informal evaluations and/or multidisciplinary consensus diagnoses (Davidson & Harrison, 2000). The lack of appropriate communication- and language-related assessment tools for children with VI further limits the level of evidence that studies can achieve.

### Risk of bias within and across studies

The PRISMA statement (Moher *et al.*, 2009) regards the assessment of risk of bias as one of the key characteristics of a systematic review as biases pose a threat to the validity of a review. The selected articles were assessed according to the identified criteria (Table 2). It was not always possible to identify if the criteria for the assessment of risk of bias were met, as explicit statements were not found in the articles. The conditions that could not be reliably labelled as absent or present were therefore recorded as ‘unclear’ instead.

**TABLE 2:** Six criteria for risk of bias within and across studies.

Source	Steps taken to avoid selection bias	Blinding of participants (1), personnel (2) or outcome assessors (3)	Control groups or tools (*)	More than one clinician involved in evaluations	Inter-rater agreement achieved	Validity (a), internal item consistency (b) and/or reliability testing (c)	Possible bias identified in the selected study
Dammeyer, 2012	✓	Unclear	Unclear	✓	Unclear	Unclear	Small sample size. Comparisons made between the difficulties common between the syndromes.
Peltzer-Karpf, 2012	Unclear	Unclear	✓	Unclear	Unclear	Unclear	Unclear
Funnell & Wilding, 2011	Single case study	Unclear	✓	✓	Unclear	Unclear	Unclear
Absoud *et al.*, 2011	✓	✓ (3)	✓ (*)	✓	X consensus rating used	✓ (a) ✓ (b) ✓ (c)	Referral pattern used. Small sample size. Variation in participant group size.
Parr *et al.*, 2010	✓	✓ (3)	✓	✓	Unclear	Unclear	Retrospective nature of the study as the true rate of ASD in the sample may be higher because ASD knowledge has developed since 1977. The impact of individual differences in environmental experience and input was not assessed.
Hoevenaars-van den Boom *et al.*, 2009	✓	✓ (3)	✓	✓	✓	✓ (b)	Difficulty diagnosing ASD. Small sample size. Diverse aetiologies of VI in participants. Adjustment for behaviour was limited due to the standardisation of the assessment. Psychometric properties of the assessment procedures required further testing.
Peltokorpi & Huttunen, 2008	Unclear	✓ (3)	✓ (*)	✓	✓	Unclear	Modifications were required for both analysis methods due to the participants’ VI. Short duration of the sample of behaviour for each child may only reveal some features of communication. Testing with multiple partners in different environments is required.
Rattray & Zeedyk, 2005	✓	✓ (3)	✓	✓	Unclear	✓ (c)	Unclear
Ashkenazy *et al.*, 2005	✓	Unclear	✓	✓	Unclear	Unclear	Small sample size. Some children in the control group may have developed difficulties later, after the upper age limit of the study.

According to Table 2, Hoevenaars-van den Boom *et al.* (2009) was the most unbiased study and met all the selected criteria for assessment of bias (Higgins *et al.*, 2011). Peltzer-Karpf (2012) met the least measures of bias, as ‘control groups’ was the only criterion identified in the article. In Dammeyer (2012) only two of the six criteria were identified. Assessment criteria for bias not met does not necessarily imply bias, but rather that the description and rationale of methodological procedures were unclear. All but one article, Dammeyer (2012), clearly stated the use of some form of control and all the studies, except Peltzer-Karpf (2012), mentioned the involvement of more than one clinician during the evaluations. Most of the selected studies, except Funnell and Wilding (2011), Peltzer-Karpf (2012), and Rattray and Zeedyk (2005), provided statements on possible biases in their studies. Seven of the selected articles (*n* = 9) met at least three out of six criteria, which indicates that risk of bias is being considered in recent research.

### Language and communication characteristics of young children with VI

By means of a thematic analysis (Appendix A) the language and communication characteristics of children with VI were identified from recent research. The studies by Parr *et al.* (2010) and Peltokorpi and Huttunen (2008) were allocated to both main themes as they discuss early intervention, ASD and multiple disabilities. The first main theme identified in analyses of the nine studies was *early intervention* (Appendix A). The early developmental difficulties described in the studies highlighted a need to support caregivers and children with VI during early stages of language and communication development.

The study by Dammeyer (2012) identified possible early predictors for language delays in children with VI. Delayed walking was associated with cognitive and language delays in participants with CHARGE syndrome. The study also found that difficulties in vision, hearing and motor skills have a compounding effect on language, and cognitive and social development in participants with Usher and CHARGE syndromes (*ibid*). It appears that the more severe the multidisabilities, the greater the impact on the participant’s development.

By means of longitudinal neuroimaging studies, Peltzer-Karpf (2012) identified that language acquisition follows the same pattern for participants with VI as for sighted controls, but that the progression is slower. With age and maturity the initial gap between the participant with VI and the participant with normal vision diminished. The author found that language delays were more prominent in the early stages of development. Multifaceted training programs focused on developmental progression instead of age-matched abilities were recommended from an early age to help overcome this initial delay sooner and to optimise neuroplasticity (*ibid*).

Funnell and Wilding (2011) showed that phonology and articulation development were not impacted by the VI, but receptive and expressive language delays were evident from the age of two years when preschool assessments commenced. Peltokorpi and Huttunen (2008) found that language and communication were impacted from the preverbal stages in children with CHARGE syndrome. This delay resulted in limited intentional communication with a greater dependency on gestures and protesting. The researchers recommended that early intervention be based on parent-child interaction. Parents that are competent and adaptive communication partners help to support communication development (Funnell & Wilding, 2011). Parr *et al.* (2010) found that basic form vision in children with optic nerve hypoplasia (ONH) and septo-optic dysplasia (SOD) is insufficient to support the development of early social and communication skills.

Rattray and Zeedyk (2005) identified three non-visual, alternative communication means to maintain the quality of communication interactions between mothers and their young children with VI. Touch, vocalisations and facial orientation are recommended to help mothers fulfil their important role in language acquisition in children with VI. All the mothers in the study instinctively used active touch and increased vocalisations as modes of communication. Children with VI used active touch during shared attention as a tactile form of communication. Although still a means of communication, the rate of vocalisation was affected by the presence of VI in the mother or child. All the mothers and children made use of facial orientation during shared attention but to a lesser degree than touch and vocalisations, indicating that facial orientation is not as important as an alternative communication means (Rattray and Zeedyk 2005).

Lastly, Ashkenazy *et al.* (2005) found that the emotional and behavioural status of children with VI impacts on their receptive and expressive language abilities. Early identification and treatment of emotional and behavioural problems in children with VI is therefore important, as delayed language development may be ameliorated.

Recent research therefore suggests that children with congenital VI show the greatest delays in the early stages of development when language and communication acquisition are more dependent on visual input. The greater the degree of VI, the more likely children with VI are to present with early social and communication difficulties (Parr *et al.*, 2010). It could be that during the early years, when children are dependent on caregivers for language development, parents may not be aware of how to adapt their interactions to stimulate development through alternative, non-visual means. It is clear that speech-language therapists need to play a greater role in early intervention for children with VI.

The second main theme identified (Appendix A) was *ASD and multiple disabilities*. Absoud *et al.* (2011) ascribed the high rate of children with VI that present with social communication difficulties and ASD to multiple factors, including visual status, age, gender, and psychological and neurological functioning. By developing an observation instrument for early accurate identification of social communication difficulties and ASD in preschool children with VI, early intervention strategies can be implemented (Absoud *et al.*, 2011). Parr *et al.* (2010) found that children with VI due to ONH and SOD, especially in the presence of significant cognitive impairment and/or profound VI, are also at risk of ASD. There was, however, no evidence that additional neuroanatomical abnormalities, other than those associated with ONH and SOD, further increased the risk of ASD. According to Parr *et al.* (2010), the co-occurrence of VI and ASD in a child significantly affects receptive and expressive language abilities. The authors offer an explanation that ASD may result as secondary condition to VI due to sensory deprivation, hormonal influences or genetic factors, but these mechanisms require further investigation (*ibid*).

Peltokorpi and Huttunen (2008) state that children with CHARGE syndrome frequently demonstrate ASD traits. Contributing factors to this behaviour may be reduced parent-child interaction due to long periods of hospitalisation and poor health, first smiles emerge later in children with CHARGE syndrome, which, with possible facial paralysis, can affect non-verbal communication, and the children display more stereotyped behaviour than other children with deafblindness (*ibid*). However, children with CHARGE syndrome demonstrated better language and communication abilities than children with ASD (*ibid*).

According to Hoevenaars-van den Boom *et al.* (2009), ASD can be overdiagnosed and mistreated in children with deafblindness due to similarities in behaviour, especially when intellectual disability co-occurs. Despite the tendency of overdiagnosis, there appears to be a high prevalence of ASD in children with deafblindness. Communication and language are the main areas affected by congenital deafblindness and children often remain at a preverbal stage (Hoevenaars-van den Boom *et al.* 2009). The co-occurrence of visual impairment, intellectual disability and ASD has a compounding effect. Children with ASD, deafblindness and intellectual disability show greater difficulty with communication functions, pragmatic skills, transitioning, problem solving, play and stereotyped behaviour than in the absence of ASD (Hoevenaars-van den Boom *et al.* 2009). Therefore, social interaction and communication skills should guide the diagnosis of ASD in children with deafblindness. ASD symptoms may present in children with deafblindness due to extreme isolation from people and the environment. The inclusion of pragmatic skills across multiple studies may be because the early development of these abilities depends heavily on vision (Dale & Salt, 2008).

The results of the systematic review confirm the observation by Chen (2001) that a characteristic of children with VI is that there are almost always associated conditions, which complicates diagnosis and management. According to House and Davidson (2000), speech-language therapists may manage children with VI in the same way as they would treat children with hearing loss, as it is the sensory difficulty that they are most familiar with. However, the presence of multiple disabilities, such as deafblindness, has an accumulated effect on language and communication development as both visual and hearing input are limited. These complex difficulties require the use of different approaches and techniques to stimulate language and communication abilities.

The participants with VI in the studies by Funnell and Wilding (2011), Hoevenaars-van den Boom *et al.* (2009), Peltokorpi and Huttunen (2008), and Peltzer-Karpf (2012) all presented with language and communication difficulties. In the study by Parr *et al.* (2010), 58% of the participants presented with at least one social, communication and/or restrictive or repetitive behaviour. The studies by Absoud *et al.* (2011), Ashkenazy *et al.* (2005), and Rattray and Zeedyk (2005) described language and communication characteristics, but did not state how many of the participants demonstrated difficulties. In the study by Dammeyer (2012), 15 of the 26 participants with Usher syndrome and three of the 17 participants with CHARGE syndrome presented with little or no language delay or intellectual disability. This may be because of the relationship between intellectual ability and language competence (Dammeyer (2012). In summary, language and communication difficulties were common in the participants of the study selection.

An identified study limitation of the systematic review may be inclusion of participants that were older than five years. During the database searches, filters were set for the age range of birth to five years old, as identified by the inclusion criteria. However, participants that were older were included in the selected studies due to intellectual disabilities.

## Conclusion

The finding that no studies were identified from developing countries, points to a great research need. Of the approximate 19 million children with VI (birth to 14 years) worldwide (WHO, 2014), an estimated 23% are blind and live in the developing region of sub-Saharan Africa (Kello & Gilbert, 2003). South African speech-language therapists can expect to encounter children with VI more often than therapists in developed countries. The children’s profile of VI and associated disorders and delays may also be different from those living in developed countries. The aetiology of the VI in developing countries is mostly acquired due to a lack of resources combined with stressful environments (Gilbert & Foster, 2001), while participants in seven of the nine selected studies presented with congenital VI. It appears that recent research is not yet investigating the communication and language development of children in developing countries with acquired VI.

The lack of appropriate assessment tools for children with VI may also limit research on the developmental characteristics of this population. The trend in research should be towards developing appropriate assessment tools, such as the studies by Absoud *et al.* (2011) and Hoevenaars-van den Boom *et al.* (2009). Following on improved assessment measures, effective language and communication stimulation techniques should be developed for caregivers of children with VI to use during the difficult early stages of development.

Investigating the language and communication difficulties of young children with VI is challenging. The visual system is the most complex sensory system, but the least mature at birth (Glass, 2002). Thus, the causes and effects of VI are numerous and intricate (Holte *et al.*, 2006). Conducting research with this diverse population is complicated, especially with the common co-occurrence of other conditions.

Based on this systematic review, there is recent evidence on the early language and communication difficulties of children with VI. However, intellectual disability, ASD and multiple disabilities do interfere with the identification of language and communication difficulties in children with VI. Six of the nine articles (Absoud *et al.*, 2011; Dammeyer, 2012; Funnell & Wilding, 2011; Hoevenaars-van den Boom *et al.*, 2009; Parr *et al.*, 2010; Peltokorpi & Huttunen, 2008) attempt to address this problem. The impact of VI itself on communication remains unclear because the effect of VI on language and communication development cannot yet be separated from the primary conditions.

Language and communication development in children with VI is not a large or popular research field in speech-language therapy. Therefore, the carryover of research into clinical practice may be limited, resulting in undertreatment and underestimation of the language and communication difficulties in young children with VI. The language and communication developmental characteristics revealed in this systematic review may assist speech-language therapists to build up a knowledge base for participation in early intervention for young children with VI and their families. To add to this knowledge base, future research needs to focus on describing the language and communication developmental characteristics of children with acquired VI in developing countries, especially within sub-Saharan Africa.

## Appendixes

**Table:** APPENDIX A: Summary of main themes, sub-themes and study outcomes relating to communication and language characteristics of young children with VI.

Main themes	Article	Sub-themes	Study outcomes
Early intervention	Dammeyer, 2012	Language abilities Congenital VI	No formal test for the language evaluation of children with deafblindness. Language delay was estimated using informal procedures and a rating scale 15 (n26) children with Usher presented with little or no language delay 3 (n17) children with CHARGE presented with little or no language delay Late age of walking may be an early predictor for: - cognitive and language delays in CHARGE syndrome - cognitive delay in Usher syndrome The combination of VI, hearing loss and delayed motor skills provided additional barriers for language, cognitive and social development. There was a correlation between the degree of deafblindness and the language delay in Usher. There was a correlation between the degree of intellectual disability and language delay in Usher and CHARGE.
		Lack of assessment tool Cognitive impairment Multiple disabilities	
	Peltzer-Karpf, 2012	Language abilities Pragmatic skills Congenital VI Neuroimaging	In children with congenital VI, the visual areas of the brain are used for non-visual tasks, such as auditory language processing. Language acquisition in children with sensory impairment follows the same overall pattern to sighted or hearing children in terms of macrostructural changes, but various subsystems, within vision, hearing, language and attention, are selectively affected. Therefore there are time lags that are most evident in the early stages of development. Development of neural systems for syntax takes longer than systems for semantics. Due to the absence of lip reading, there is extended sound sorting and delays in phonological learning. Congenital VI results in the lack of referential gaze, which causes slower concept formation. This affects morphological and syntactic development. Initially, the single-word stage is delayed, but this is followed by intense lexical acceleration rate.
			Language delay decreases with age and maturity, resulting in developmental profiles process-oriented and not age-matched. Interdisciplinary, process-oriented research helps to apply multifaceted training programs as early and efficiently as possible to optimise children’s development
	Funnell & Wilding, 2011	Language abilities Speech production	Language delay identified from the age of two years. Progressive receptive and expressive language delay over the years. Phonology and articulation were normal as the systems are not dependent on vision. Severely impaired visual object naming contrasted with normal understanding of the spoken names of objects.
	Parr *et al.*, 2010	Pragmatic skills Language abilities Congenital VI Lack of assessment tool ASD Multiple disabilities	Standard measures of social communication development and ASD are not available for young children with VI.
			There was at least one SCRR difficulty in 48 (n83) of the participants. 37% of the sample had difficulties in all three domains. Children with one or more SCRR and ASD have a developmental quotient within the learning difficulty range when compared to norms of children with VI. Basic form vision is not sufficient to support early social and communication development in children with ONH and SOD.
	Peltokorpi & Huttunen, 2008	Communication abilities Language abilities Pragmatic skills Congenital VI Stereotyped behaviour Multiple disabilities Parent-child interaction Tool modification required	Communication was impacted from the preverbal stage due to deafblindness, hospitalisation and facial paralysis.
			Children with CHARGE demonstrate more stereotypical behaviour than other children with deafblindness.
			All the children (n3): - used mainly gestures - made initiations slightly under half of the total number of communication expressions, indicating active involvement - used eye contact but limited even though sight was used to explore toys - showed limited requesting - protesting was the most common communication function. Intentional communicative acts were present in all three participants, but the frequency was low compared to the total number of communicative acts. Children with multiple disabilities demonstrate only some intentional communication in early stages of language development.
			Careful examinations of the communicative behaviour between a child and parent can serve as a basis for early intervention.
			Atypical features of visual behaviour make interpreting communication challenging. Audiological management is important for the development of communication and language in children with CHARGE.
	Rattray & Zeedyk, 2005	Communication abilities Communication means Pragmatic skills Parent-child interaction	All mothers used activ etouch as a mode for communication, but mothers of children with VI used increased active touch before gradually decreasing it. All mothers and infants showed more active and passive touch during shared attention, indicating that touch is a communication means. Active touch is prominent in children with VI as a tactile form of communication due to the lack of visual communication during shared attention. All mothers and children used increased vocalisations during joint attention as a means of communication.
			VI, of mother or child, may affect the overall rate of vocalisation, but is still used as a means of communication.
			All mothers and children used facial orientation during shared attention, but less than touch and vocalisations, indicating that facial orientation is not as important as a communication means. VI itself does not automatically decrease the quality of communication interactions between mothers and infants, but does necessitate the reliance on alternative, non-visual communication means. The VI status of the mother and/or child impacts the communication interaction. Mothers have an important role in children’s communication acquisition.
	Ashkenazy *et al.*, 2005	Language abilities Emotional status Parent-child interaction Congenital VI	Receptive language attainments were significantly affected by the child’s emotional and behavioural status.
			The interaction between the child’s age and the mother’s level of education impacts on receptive language: older children of mothers with less education show compromised receptive language abilities. Expressive language attainments were associated with the child’s emotional and behavioural status and not significantly with the mother’s level of education. There was a strong association between development and a child with VI’s emotional and behavioural status.
			Early identification and treatment of emotional and behavioural problems lead to better emotional status and thus improved development

**APPENDIX A (Continued):** Summary of main themes, sub-themes and study outcomes relating to communication and language characteristics of young children with VI.

Main themes	Article	Sub-themes	Study outcomes
ASD and multiple disabilities	Absoud *et al.*, 2011	Pragmatic skills Congenital VI Lack of assessment tool Early intervention	There is a lack of ASD and early social communication assessments tools for children with VI.
			A high rate of children with VI present with social communication difficulties and ASD, but there is no test to confirm this.
			The development of the Visual Impairment and Social Communication Schedule (VISS) can assist in early ASD diagnosis for children with VI and subsequent appropriate early intervention.
	Parr *et al.*, 2010	Language abilities Pragmatic skills Congenital VI Stereotyped behaviour	31% of the sample received an ASD diagnosis. Significant cognitive impairment in children with ONH and SOD show a greater risk for ASD. Slightly more children with SOD where diagnosed with ASD than children with ONH. Children with PVI were more likely to present with at least one SCRR difficulty and to show all three SCRR difficulties than children with SVI, but were only slightly more likely to receive an ASD diagnosis. VI with ASD resulted in significantly lower verbal comprehension and expressive language structure. ASD was typically diagnosed in children with ONH or SOD, usually between 2.4 to 4.6 years. No evidence that additional neuro-anatomical abnormalities, other than those associated with ONH and SOD, further increased the risk of ASD
	Hoevenaars-van den Boom	Language abilities Pragmatics skills Congenital VI Stereotyped behaviour Lack of assessment tool Cognitive impairment Differential diagnosis	It is difficult to distinguish ASD from deafblindness behaviours, especially in the presence of intellectual disability, and this can lead to overdiagnosis and incorrect intervention. The presence of congenital deafblindness showed an increased risk ASD. Communication and language development were primarily affected by congenital deafblindness, although other developmental areas were likely to be impacted. People with deafblindness often remain at a prelingual communication level and may never reach a symbolic communication level, especially in the presence of intellectual disability. Children with deafblindness demonstrated shared attention, but learning and using nonverbal behaviour was compromised by VI.
			Children with deafblindness missed auditory and visual communicative signals, unlike in ASD where signals were not understood.
			The existing standardised tests, questionnaires and developmental scales for ASD are not reliable or valid for people with deafblindness because the accumulated effect of multiple disabilities is not considered. Children with congenital deafblindness had similar characteristics to the ASD triad of impairment. Children with ASD, intellectual disability and deafblindness had significantly more difficulty than children with intellectual disabilities and deafblindness but no ASD in terms of: - openness for contact - joint attention - communication functions. Children with ASD, intellectual disabilities and deafblindness had almost statistically significantly more difficulty than children with intellectual disabilities and deafblindness but no ASD in terms of: - coping with changes - problem solving strategies. Children with ASD, intellectual disabilities and deafblindness did not have significantly more difficulty than children with intellectual disabilities and deafblindness but no ASD in terms of: - stereotyped behaviour - exploration and play. The stereotyped behaviours demonstrated by children with deafblindness decreased with increased: - age - interaction initiation and maintenance - opportunity to communicate. Both children with ASD, intellectual disabilities and deafblindness, and children with intellectual disabilities and deafblindness but no ASD demonstrate: - limited functional play as this may have been linked to intellectual disabilities - increased object manipulation Children with ASD, intellectual disabilities and deafblindness had more ASD-specific behaviours than children with intellectual disabilities and deafblindness but no ASD.
			The results can assist with differentiation during diagnosis.
	Peltokorpi & Huttunen, 2008	Language abilities Communication abilities Pragmatic skills Congenital VI	Children with CHARGE demonstrated ASD-like traits, but their language and communication was better than children with ASD.
			Limited eye contact may be due to VI or ASD-like behaviour.

ASD, Autism Spectrum Disorder; VI, visual impairment; OHN, optic nerve hypoplasia; SOD, septo-optic dysplasia; SCRR, social, communication and/or restrictive or repetitive behaviour; PVI, profound visual impairment; SVI, severe visual impairment.
